# Pseudohyponatremia Caused by Severe Hemolysis

**DOI:** 10.7759/cureus.84824

**Published:** 2025-05-26

**Authors:** Hitoshi Yonemoto

**Affiliations:** 1 Infectious Disease, Yamato Takada Municipal Hospital, Yamatotakada, JPN

**Keywords:** hemolysis, hyponatremia, ion-selective electrode, pseudohyponatremia, serum free hemoglobin

## Abstract

A 78-year-old man with influenza presented to the hospital. Initial blood tests revealed severe hemolysis accompanied by a decreased serum sodium concentration. However, repeat testing showed no hemolysis, and the serum sodium level was within the normal range. Notably, the serum sodium concentration in the initial sample, when measured using a blood gas analyzer, was also normal. These findings led to a diagnosis of pseudohyponatremia secondary to severe hemolysis. The underlying mechanisms of this condition are discussed herein.

## Introduction

Hemolysis can result from both in vivo and in vitro factors, significantly impacting the accuracy and reliability of clinical laboratory tests. It is observed in approximately 3-12.4% of blood samples submitted from emergency departments [1], with the majority attributed to in vitro causes, such as suboptimal blood collection techniques. While it is well established that hemolysis leads to elevated levels of lactate dehydrogenase (LDH), potassium, and aspartate aminotransferase (AST), a range of other laboratory parameters may also be affected, including a typically decreased serum sodium concentration [2]. Pseudohyponatremia is a known laboratory artifact that arises when serum protein or lipid concentrations are abnormally high [3]. If unrecognized, this condition can lead to inappropriate treatment aimed at other causes of hyponatremia, potentially resulting in adverse clinical outcomes. This case represents the first reported instance of pseudohyponatremia caused by severe hemolysis, and the underlying mechanism is discussed.

## Case presentation

A 78-year-old man presented to his primary care physician with complaints of fever and cough that had started the previous day. He also reported fatigue and lower-limb weakness. A rapid diagnostic test was positive for influenza A, and he was subsequently referred to this hospital for further evaluation.

The patient had a 20-pack-year smoking history but had quit smoking 30 years prior to the current presentation. His medical history included chronic obstructive pulmonary disease, allergic rhinitis, hyperuricemia, glaucoma, and a history of gastric ulcer surgery. His regular medications included a combination inhaler of vilanterol and fluticasone, a leukotriene receptor antagonist, and febuxostat.

On examination, he was fully conscious and not in respiratory distress. His body temperature was 37.1°C, blood pressure 172/96 mm Hg, pulse rate 79 beats per minute, and oxygen saturation 95% on room air. No edema was noted in the back or extremities.

Initial blood tests revealed severe hemolysis and mild lipemia. The laboratory findings are presented in Table 1. Levels of LDH, potassium, and AST were markedly elevated, while the serum sodium concentration was decreased, indicating hyponatremia. Anemia and thrombocytopenia were absent. Venous blood gas analysis from the same sample showed a normal serum sodium concentration, no evidence of hypercapnia, and a normal blood glucose level. While elevated LDH, potassium, and AST levels are typical findings in hemolyzed samples, hyponatremia is less commonly observed. Syndrome of inappropriate antidiuretic hormone secretion associated with influenza was considered as a differential diagnosis.

**Table 1:** Summary of laboratory data

Variable	Reference range	Initial blood draw	Second blood draw
White cell count (per μL)	3,300-8,600	6,700	N/A
Hemoglobin (g/dL)	13.7-16.8	14.9	N/A
Hematocrit (%)	40.7-50.1	43.7	N/A
Platelet count (per μL)	158,000-348,000	192,000	N/A
Aspartate aminotransferase (U/L)	13-30	327	35
Alanine aminotransferase (U/L)	8-42	51	23
Alkaline phosphatase (U/L)	38-113	76	79
Total bilirubin (mg/dL)	0.4-1.5	0.59	0.55
Lactate dehydrogenase (U/L)	124-222	2,404	169
Creatine kinase (U/L)	59-248	193	185
Total protein (g/dL)	6.6-8.1	7.1	6.8
Albumin (g/dL)	4.1-5.1	3.6	3.7
Urea nitrogen (mg/dL)	8.0-20.0	12.2	12
Creatinine (mg/dL)	0.65-1.07	0.65	1.01
Sodium (mEq/L)	138-145	127	138
Potassium (mEq/L)	3.6-4.8	13.9	3.6
Chloride (mEq/L)	101-108	97	101
Blood gas sodium (mmol/L)	135-148	137.7	N/A
Blood gas pCO₂ (mm Hg)	N/A	38	N/A
Blood gas glucose (mg/dL)	66-93	112	N/A
Serum appearance	N/A	Severely hemolyzed, mildly lipemic	Mildly lipemic

To confirm the findings, a repeat blood sample was collected, which showed no hemolysis and only mild lipemia. LDH and potassium levels were within normal ranges, and AST was nearly normal. The serum sodium concentration was also normal, contrasting with the initial measurement (Table 1). These findings suggested that the initial abnormal values were due to in vitro hemolysis, and the initial hyponatremia was identified as pseudohyponatremia caused by elevated serum hemoglobin.

The patient was prescribed oseltamivir and inhaled procaterol and was subsequently discharged home. His condition improved thereafter.

## Discussion

Pseudohyponatremia is a laboratory artifact that occurs when blood concentrations of proteins or lipids are abnormally elevated [3]. It has been associated with various conditions, including hyperproteinemia, hypertriglyceridemia, and hypercholesterolemia [3]. Hyperproteinemia, in turn, may arise from monoclonal gammopathy, HIV infection, or intravenous immunoglobulin administration [3-6]. One study has investigated the relationship between experimentally induced hemolysis and hyponatremia, as well as the mechanisms involved [7]. However, to date, no published case report has documented pseudohyponatremia caused by hemolysis in a patient.

In hemolysis, rupture of red blood cell membranes leads to the release of intracellular components such as hemoglobin into the plasma. Although serum free hemoglobin concentration was not measured in this case, based on existing literature correlating the degree of hemolysis with laboratory changes, an LDH level of 2,404 U/L suggests serum free hemoglobin concentrations ranging from 10.3 to 20.6 g/L [2]. Compared with normal serum, this implies a significantly increased protein load. However, in the present case, the total serum protein level did not increase to a degree consistent with those estimations. Few studies have explored the relationship between serum free hemoglobin and total protein concentration, and although the reason for this discrepancy remains unclear, methodological differences between assays may be a contributing factor. In previously reported cases of pseudohyponatremia caused by hyperproteinemia, total serum protein concentrations often exceeded 10 g/dL, which is higher than observed in this case. However, the degree of hyponatremia in those reports was also more severe [4-6].

Most modern clinical laboratories measure serum sodium using ion-selective electrode (ISE) methods, which include both direct and indirect techniques. Indirect ISE is used in most automated analyzers, while direct ISE is employed in blood gas analyzers. Serum consists of solid content, primarily proteins and lipids, and water. Normally, serum water content (SWC) accounts for 93% of the volume, with solid content (SSC) making up the remaining 7%. In the indirect ISE method, a fixed volume of diluent is added to the sample to reduce the required blood volume for measurement. Sodium concentration is measured in the diluted serum water, and the result is multiplied by the dilution factor to estimate the serum sodium concentration. Since sodium is present only in the water phase, any increase in SSC reduces the proportion of SWC. This electrolyte exclusion effect leads to an underestimation of sodium concentration when the actual dilution factor is used. Automated analyzers, however, assume a constant 93% SWC, which can result in an underestimated dilution factor and a falsely low sodium reading (dilution effect).

Aziz et al. [3] identified three mechanisms responsible for pseudohyponatremia when using indirect ISE: electrolyte exclusion, dilution effect, and hyperviscosity. The hyperviscosity effect occurs when increased sample viscosity impairs the transfer of serum (but not the diluent) to the measurement chamber, resulting in underestimation of sodium concentration. In contrast, direct ISE methods used in blood gas analyzers do not involve sample dilution and are unaffected by these artifacts, thereby providing a more accurate measurement of serum sodium.

In this case, the serum sodium concentration measured by the blood gas analyzer was 137.7 mmol/L, consistent with the 138 mEq/L result obtained from the second (non-hemolyzed) sample analyzed by the automated clinical chemistry analyzer. These values likely reflect the true serum sodium concentration.

Serum osmolality, triglycerides, cholesterol, and serum free hemoglobin were not measured in this case. Thus, the diagnosis of pseudohyponatremia remains presumptive, based on indirect evidence. Nevertheless, the presence of comparable lipemia in both samples, the normalization of sodium levels in the second, non-hemolyzed sample, and the patient’s euvolemic status all strongly suggest that the hyponatremia observed in the first blood draw was due to pseudohyponatremia caused by hemolysis. The sodium concentration obtained from the blood gas analyzer further supports this conclusion.

## Conclusions

The treatment of hypotonic hyponatremia, the most common type, differs substantially from that of pseudohyponatremia. If pseudohyponatremia is not promptly recognized, treatment interventions aimed at correcting sodium levels may be inappropriate and potentially harmful. When hyponatremia is observed in a severely hemolyzed sample, repeat testing should be performed to rule out pseudohyponatremia due to in vitro hemolysis. In addition, the serum sodium concentration measured using a blood gas analyzer should be reviewed to aid accurate diagnosis.
